# Cu and Zn isotope ratio variations in plasma for survival prediction in hematological malignancy cases

**DOI:** 10.1038/s41598-020-71764-7

**Published:** 2020-10-02

**Authors:** Agustina A. M. B. Hastuti, Marta Costas-Rodríguez, Akihiro Matsunaga, Takayuki Ichinose, Shotaro Hagiwara, Mari Shimura, Frank Vanhaecke

**Affiliations:** 1 Department of Chemistry, Atomic & Mass Spectrometry – A&MS Research Unit, Ghent University, Campus Sterre, Krijgslaan 281 – S12, 9000 Ghent, Belgium; 2 Department of Intractable Diseases, Research Institute, National Center for Global Health and Medicine, 1-21-1 Toyama, Shinjuku, Tokyo, 162-8655 Japan; 3 Inorganic Analysis Laboratories, Toray Research Center, Inc., Otsu, Shiga 520-8567 Japan; 4 Division of Hematology, Internal Medicine, Hospital, National Center for Global Health and Medicine, Shinjuku, Tokyo Japan; 5 Present Address: Department of Hematology, Tokyo Women’s Medical University, Shinjuku, Tokyo 162-8666 Japan

**Keywords:** Haematological cancer, Metals, Analytical chemistry, Bioanalytical chemistry, Mass spectrometry, Biomarkers

## Abstract

We have examined potential changes in the isotopic compositions of Fe, Cu and Zn (using multi-collector inductively coupled plasma-mass spectrometry) and the corresponding concentrations (using inductively coupled plasma-atomic emission spectrometry) in plasma from hematological malignancy (HM) patients and assessed their prognostic capability. Together with clinical laboratory test values, data were examined in view of a 5-years survival prediction. Plasma Cu and Zn isotope ratios and their concentrations were significantly different in HM patients compared to matched controls (*P* < 0.05). Both δ^65^Cu and δ^66^Zn values showed significant mortality hazard ratios (HRs) in HM. The group of patients with decreased δ^65^Cu and increased δ^66^Zn values showed significantly poorer survival from the early phase (HR 3.9; *P* = 0.001), forming a unique cohort not identified based on laboratory test values. Well-known prognostic factors for HM, such as the creatinine level, and anemia-related values were highly correlated with the δ^66^Zn value (*P* < 0.05). Time-dependent ROC curves based on the δ^65^Cu or δ^66^Zn value were similar to that based on the creatinine concentration (a well-known prognostic factor in HM), indicating that δ^65^Cu or δ^66^Zn values are useful for prognosis of HM. Variations in stable isotope ratios of essential mineral elements have thus been shown to reflect alterations in their homeostasis due to physiological changes in malignancies with higher sensitivity than concentrations do.

## Introduction

Essential metals play a pivotal role in numerous biological processes, especially for maintaining protein structure and in cellular biochemical reactions. For redox-active elements, such as Cu and Fe, the electron transfer between the reduced and the oxidized states is exploited for catalysis of protein activities, such as energy production and oxidative processes^1^. As a result of its redox activity, such a metal can also damage proteins, lipids, nucleic acids, and other cellular compounds when present in excess. Zinc, a redox-inert element, is present in one-tenth of the proteins in the human body and participates in many cellular signalling cascades^2^.

Alteration of the concentrations of Cu and Zn in serum has been widely described in cancer, particularly in the case of hematologic malignancies (HMs). HMs can be roughly classified into leukemia, lymphoma, and myeloma; while all these diseases affect the hematopoietic system, they show diverse features. HM, as well as other cancer types, has been associated to increased oxidative stress, which can be related to Cu toxicity^3^ and/or reduced activity of Cu/Zn superoxide dismutase (SOD)^4^. Cu/Zn SOD is a free radical scavenger, the role of which is maintaining a low level of reactive oxygen species. A meta-analysis on acute leukemia demonstrated that the serum Cu concentration is significantly higher, while that of Zn is significantly lower in patients than in controls^5^. This pattern for Cu and Zn serum concentrations was also observed in a meta-analysis of bladder cancer^6^ and was also associated with an increased risk of oral cancer^7^. Other studies also found serum Cu and/or Zn concentrations deviating from the control range, but without following this specific pattern^5, 6, 8^. Despite the large amount of epidemiological data linking metals to HM, their clinical impact on the disease remains unclear. Metal concentrations in serum are tightly controlled, but they vary widely among individuals as they are influenced by many parameters unrelated to either element status or cancer, e.g., the presence of infection, inflammation, age, gender, diet, smoking, etc^9^.

High-precision isotopic analysis has been shown to be a suitable tool for detecting alterations in metal homeostasis due to physiological changes^10, 11^, also those related to cancer. The serum and whole blood Cu isotopic compositions have been shown to be significantly lighter (enriched in the light ^63^Cu isotope) in breast cancer, colorectal cancer^12^, and hepatocellular carcinoma patients^13^ compared to controls. Reversely, the Cu isotopic composition in tumour tissue is heavier (enriched in the ^65^Cu isotope) than that in adjacent healthy tissue^13, 14^. No differences were established in the serum and whole blood Zn isotopic composition in breast cancer^15^, colon cancer, and prostate cancer patients^11^ compared to that of controls, but the breast tumour tissue was shown to be enriched in the light ^64^Zn isotope compared to healthy tissue^15^. Metal isotopic compositions may thus reflect changes in metal homeostasis with higher sensitivity than metal concentrations do, such that high-precision isotopic analysis can detect physiological abnormalities at an early stage^12, 16^.

In this study, we examined potential changes in the isotopic compositions of Fe, Cu and Zn (using multi-collector inductively coupled plasma-mass spectrometry MC-ICP-MS) and the corresponding metal concentrations (using inductively coupled plasma-atomic emission spectrometry ICP-AES) in plasma of HM patients compared to age- and gender-matched healthy controls. The prognostic capability of the metal isotope ratios was examined via survival analyses, including mortality hazard ratios (HRs), survival curves and time-dependent ROC analysis.

## Results

### Comparison between HM patients and controls

**Figure 1:**
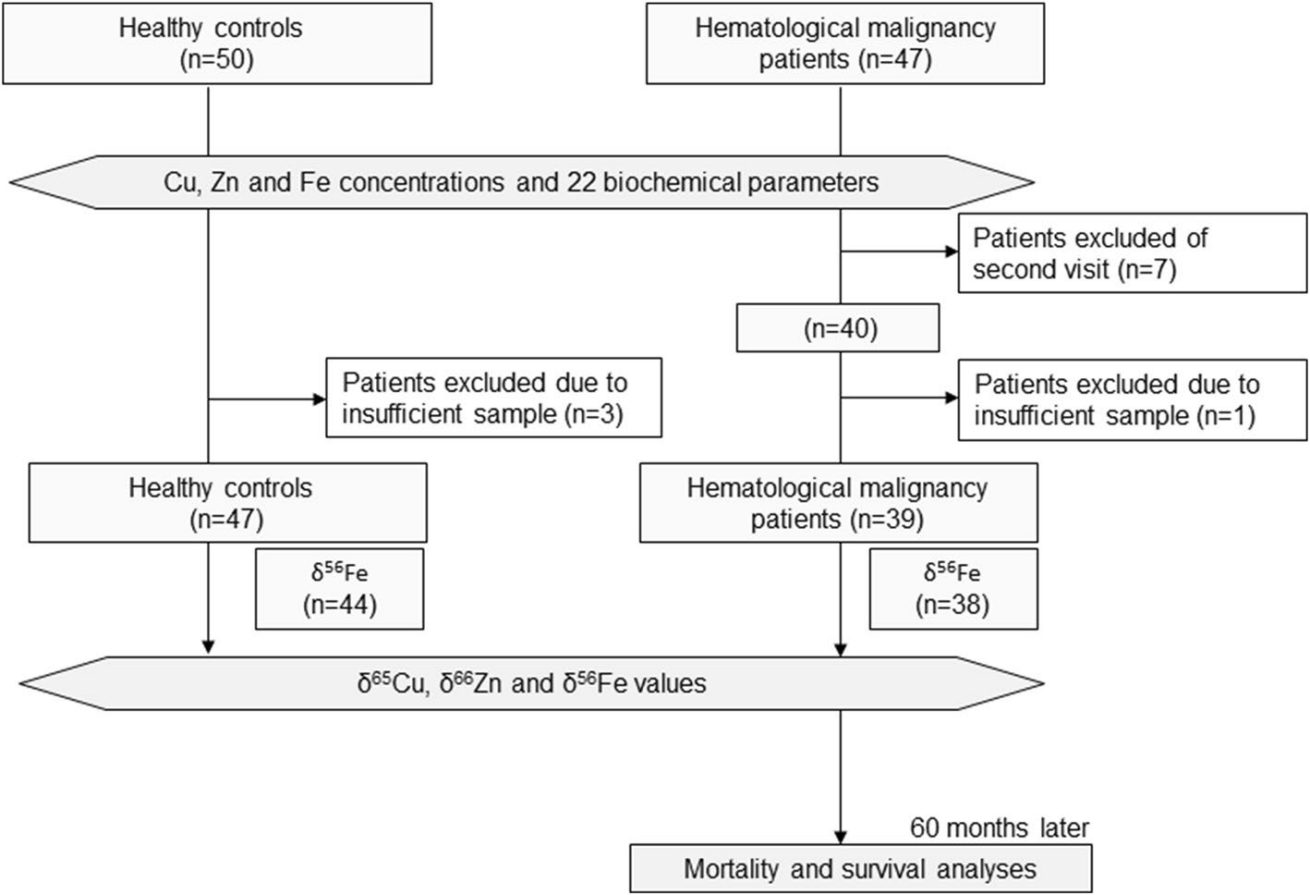
Diagram describing the selection of patient/control samples included in the study.

Patients suffering from HMs, including acute lymphoblastic leukemia (ALL), acute myeloid leukemia (AML), multiple myeloma (MM) and non-Hodgkin's lymphoma (NHL), were recruited from the hospital of the National Center for Global Health and Medicine (NCGM) in Tokyo, Japan. Patients were followed up for a period of 5 years from the first visit onwards or to death for survival analysis. As controls, gender- and age-matched healthy cases were used. Figure 1 presents the flowchart of this prospective study.

Establishing the isotopic variability within a healthy population is crucial, as the reference range might be affected by nutritional and metal status, basal metabolic rate, geographical origin, gender, age, etc. Although the Fe, Cu and Zn isotopic compositions in blood plasma, serum, red blood cells or whole blood of healthy individuals from different geographical origins have been documented, data are still scarce and furthermore, only a few works reported the isotopic composition of the three elements for the same sample/individual^17, 18^. The plasma Fe, Cu and Zn isotopic compositions for each individual are provided in Table S1. Overall, the plasma δ^56^Fe values for healthy middle-aged Japanese individuals (controls) were within the range of the serum δ^56^Fe values of Thai healthy individuals, which are characterized by a lighter plasma/serum Fe isotopic composition than reference populations from other geographical areas (Figs. S1A,B), hypothetically attributed to different dietary habits and Fe nutritional status^19–21^. The δ^65^Cu values in plasma of Japanese healthy individuals were in agreement with those reported in serum for healthy individuals from other geographical areas^16–18, 22, 23^ (Fig. S1C), while the δ^66^Zn values in plasma were slightly heavier than those in serum from healthy populations of other geographical origins (Fig. S1D), potentially associated to the sample type and/or dietary habits^24^. In this study, we used a Japanese cohort (all individuals also residing in Japan) for further analysis.

**Figure 2:**
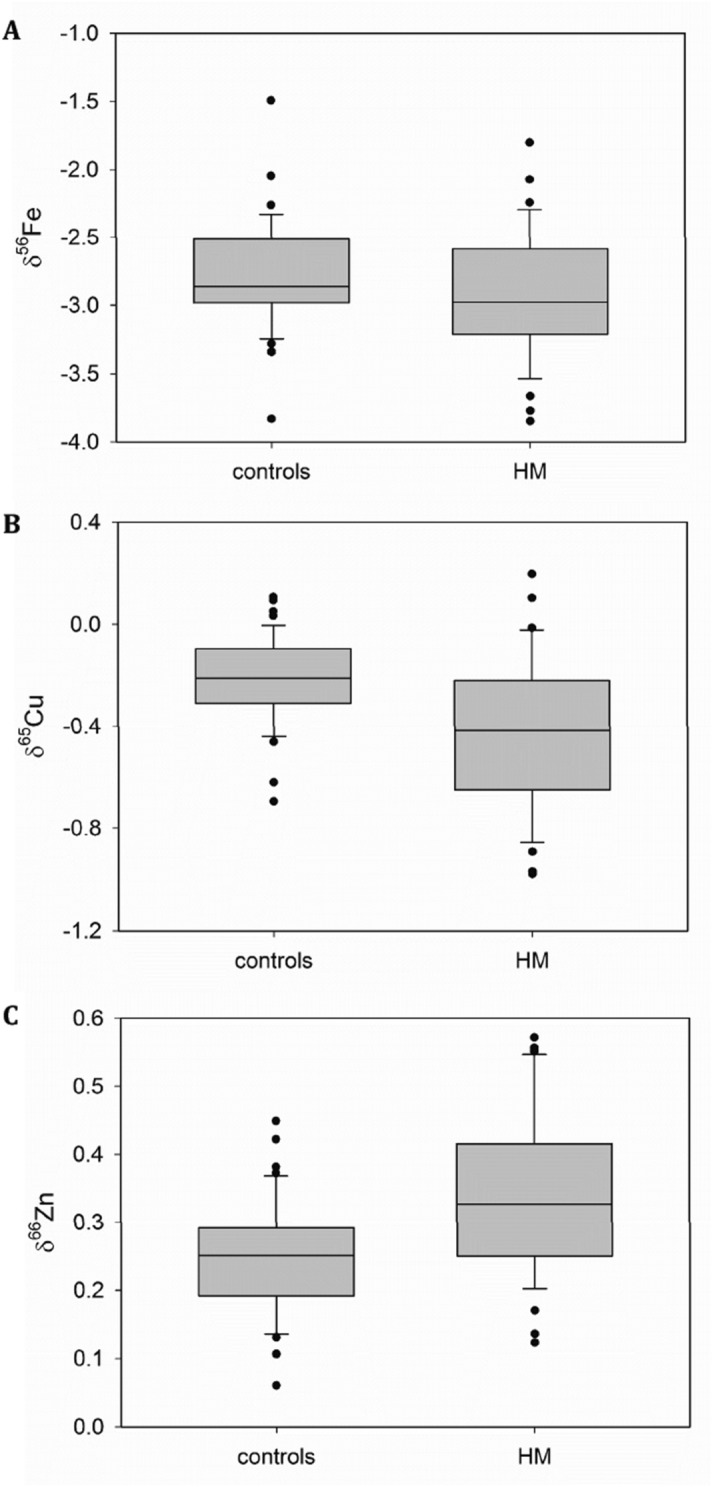
Box plots (created using SigmaPlot version 13, Systat Software, Inc, San Jose, CA, USA) for the isotopic compositions of Fe (**A**), Cu (**B**) and Zn (**C**) in plasma of HM patients and controls. These box plots compile the median, quartiles and extreme values; individual dots are outliers.

The Fe, Cu and Zn isotopic compositions, their concentrations, as well as the laboratory test values, for both the HM patients and controls are compiled in Table 1. HM patients showed plasma δ^65^Cu and δ^66^Zn values that are significantly different from those in the control group (*P* < 0.001 and *P* = 0.007, respectively), whereas the δ^56^Fe value was not significantly different (*P* = 0.09). Box plots for Fe, Cu and Zn isotopic compositions are shown in Fig. 2A–C. The plasma δ^65^Cu value tended to decrease, whereas the δ^66^Zn value tended to increase in HM patients compared to controls.

**Table 1:** Characteristics and (bio)chemical data for the HM patients and controls.

			Control		Hematological malignancy (HM)		
Sample number			50^†^		40^†^		
Age: mean (SD^‡^)			59.3 (12.2)		57.3 (15.2)		
Female/Male			25/25		19/21		
Disease (%)	ALL^††^		0 (0%)		5 (12.5%)		
	AML^††^		0 (0%)		17 (42.5%)		
	NM^††^		0 (0%)		9 (22.5%)		
	NHL^††^		0 (0%)		9 (22.5%)		

Isotope ratios			Mean (SD^‡^)	Median (ICR^‡‡^)	Mean (SD^‡^)	Median (ICR^‡‡^)	*P* value
Items		Unit					
δ^56^Fe		‰	− 2.77 (0.40)	− 2.84 (− 3.0, − 2.7)	− 2.90 (0.46)	− 3.00 (− 3.1, − 2.7)	0.096
δ^65^Cu		‰	− 0.21 (0.17)	− 0.21 (− 0.26, − 0.14)	− 0.43 (0.29)	− 0.42 (− 0.61, − 0.30)	< 0.001**
δ^66^Zn		‰	0.25 (0.08)	0.25 (0.22, 0.27)	0.34 (0.12)	0.33 (0.26, 0.39)	0.007**

Element concentrations			Mean (SD^‡^)	Median (ICR^‡‡^)	Mean (SD^‡^)	Median (ICR^‡‡^)	*P* value
Items		Unit					
Fe		× 10^–1^ µg/mL	11 (3.2)	9.6 (8.7, 11)	14 (6.1)	14 (10, 17)	0.007**
Cu		× 10^–1^ µg/mL	7.6 (1.1)	7.5 (7.1, 7.8)	11 (4.0)	9.9 (8.8, 11)	< 0.001**
Zn		× 10^–1^ µg/mL	7.1 (0.86)	7.0 (6.7, 7.5)	5.7 (1.3)	5.7 (5.3, 6.2)	< 0.001**

Laboratory tests			Mean (SD^‡^)	Median (ICR^‡‡^)	Mean (SD^‡^)	Median (ICR^‡‡^)	*P* value
Items	Abb.^††^	unit					
White blood cells	WBC	× 10^3^/mL	5.1 (1.3)	5.3 (4.7, 5.5)	47 (134)	6.3 (3.0, 0.10)	0.001**
Blasts	BLAS	× 10^3^/mL	0.0 (0.0)	0.0 (0.0, 0.0)	17 (43)	0.0 (0.0, 3.6)	< 0.001**
Neutrophils	NEU	× 10^3^/mL	3.0 (1.0)	2.7 (2.5, 3.4)	5.7 (9.2)	2.6 (1.0, 4.4)	< 0.001**
Monocytes	MON	× 10^2^/mL	0.22 (0.13)	0.21 (0.16, 0.26)	11 (66)	0.13 (0.07, 0.32)	0.17
Eosinophils	EOS	× 10^2^/mL	1.5 (1.2)	1.3 (0.76, 1.7)	0.80 (1.9)	0.0 (0.0, 0.20)	< 0.001**
Basophils	BAS	× 10^1^/mL	4.3 (4.9)	3.8 (0.0, 5.7)	1.1 (3.4)	0.0 (0.0, 0.0)	0.011
Lymphocites	LYM	× 10^3^/mL	1.7 (0.59)	1.7 (1.3, 1.9)	12 (46)	1.2 (0.81, 2.3)	0.008**
Red blood cells	RBC	× 10^5^/mL	4.7 (0.43)	4.8 (4.6, 4.9)	2.9 (0.86)	2.9 (2.4, 3.5)	< 0.001**
Hematocrit	Ht	%	44 (3.7)	44 (42, 45)	28 (7.6)	27 (23, 32)	< 0.001**
Hemoglobin	Hb	× 10^1^ g/dL	1.4 (0.14)	1.4 (1.4, 1.5)	0.93 (0.26)	0.89 (0.78, 1.1)	< 0.001**
Platelets	PLT	× 10^5^/mL	2.3 (0.53)	2.3 (2.0, 2.5)	1.2 (1.1)	0.85 (0.46, 1.6)	< 0.001**
Albumin	ALB	× 10^0^ g/dL	4.4 (0.23)	4.4 (4.3, 4.4)	3.5 (0.69)	3.7 (3.3, 3.9)	< 0.001**
Total bilirubin	TBI	× 10^0^ mg/dL	1.1 (0.36)	1.0 (1.0, 1.1)	0.82 (0.60)	0.70 (0.50, 0.80)	< 0.001**
Aspartate aminotransferase	GOT	× 10^1^ U/L	2.3 (1.1)	2.2 (1.9, 2.4)	3.2 (2.9)	2.2 (1.9, 2.7)	0.40
Alanine aminotransferase	GPT	× 10^1^ U/L	2.5 (1.7)	1.9 (1.8, 2.5)	3.0 (4.7)	1.8 (1.4, 2.3)	0.59
Lactate dehydrogenase	LDH	× 10^2^ U/L	1.9 (0.56)	1.8 (1.7, 1.9)	4.3 (4.0)	2.6 (2.4, 4.0)	< 0.001**
Blood urea nitrogen	BUN	× 10^1^ mg/dL	1.6 (0.43)	1.5 (1.4, 1.7)	1.7 (1.1)	1.4 (1.2, 1.6)	0.24
Creatinine	CRE	× 10^–1^ mg/dL	7.3 (1.7)	7.2 (6.7, 7.8)	9.1 (5.7)	7.1 (6.2, 9.0)	0.16
Uric acid	UA	× 10^0^ mg/dL	5.6 (1.3)	5.4 (4.8, 6.3)	6.4 (3.0)	5.7 (4.5, 7.0)	0.33
Sodium	Na	× 10^2^ mEq/L	1.4 (0.02)	1.4 (1.4, 1.4)	1.4 (0.06)	1.4 (1.4, 1.4)	< 0.001**
Potasium	K	× 10^0^ mEq/L	4.1 (0.32)	4.1 (4.0, 4.2)	3.9 (0.50)	3.9 (3.7, 4.2)	0.044
Chloride	Cl	× 10^2^ mEq/L	1.0 (0.15)	1.1 (1.1, 1.1)	1.0 (0.042)	1.0 (1.0, 1.0)	0.002**

Kolmogorov–Smirnov test. **P < 0.01; ^‡^SD: standard deviation; ^‡‡^ICR: inter-quartile range.

^†^Number of healthy controls was 47 for δ^65^Cu and δ^66^Zn, and 44 for δ^56^Fe.

^†^Number of HM patients was 39 for δ^65^Cu and δ^66^Zn, and 38 for δ^56^Fe.

^††^*ALL* Acute Lymphoblastic Leukemia, *AML* Acute Myeloid Leukemia, *MM* Multiple Myeloma, *NHL* Non-Hodgkin’s lymphoma, *Abb*. abbreviation.

While the concentrations of Fe and Cu were significantly elevated (by ca. 30 and 45%, respectively), the Zn concentration was ca. 20% lower in HM patients than in controls. Also, laboratory test values, which are currently used for diagnosing HMs, deviated from the reference values in a highly significant way (*P* < 0.01, Table 1). The alteration of plasma metal concentrations (elevated for Cu and reduced for Zn) and laboratory test values in HM patients are consistent with reported data^5–8, 25^.

### Five-year prognosis in HM patients using the metal isotope ratios

**Figure 3:**
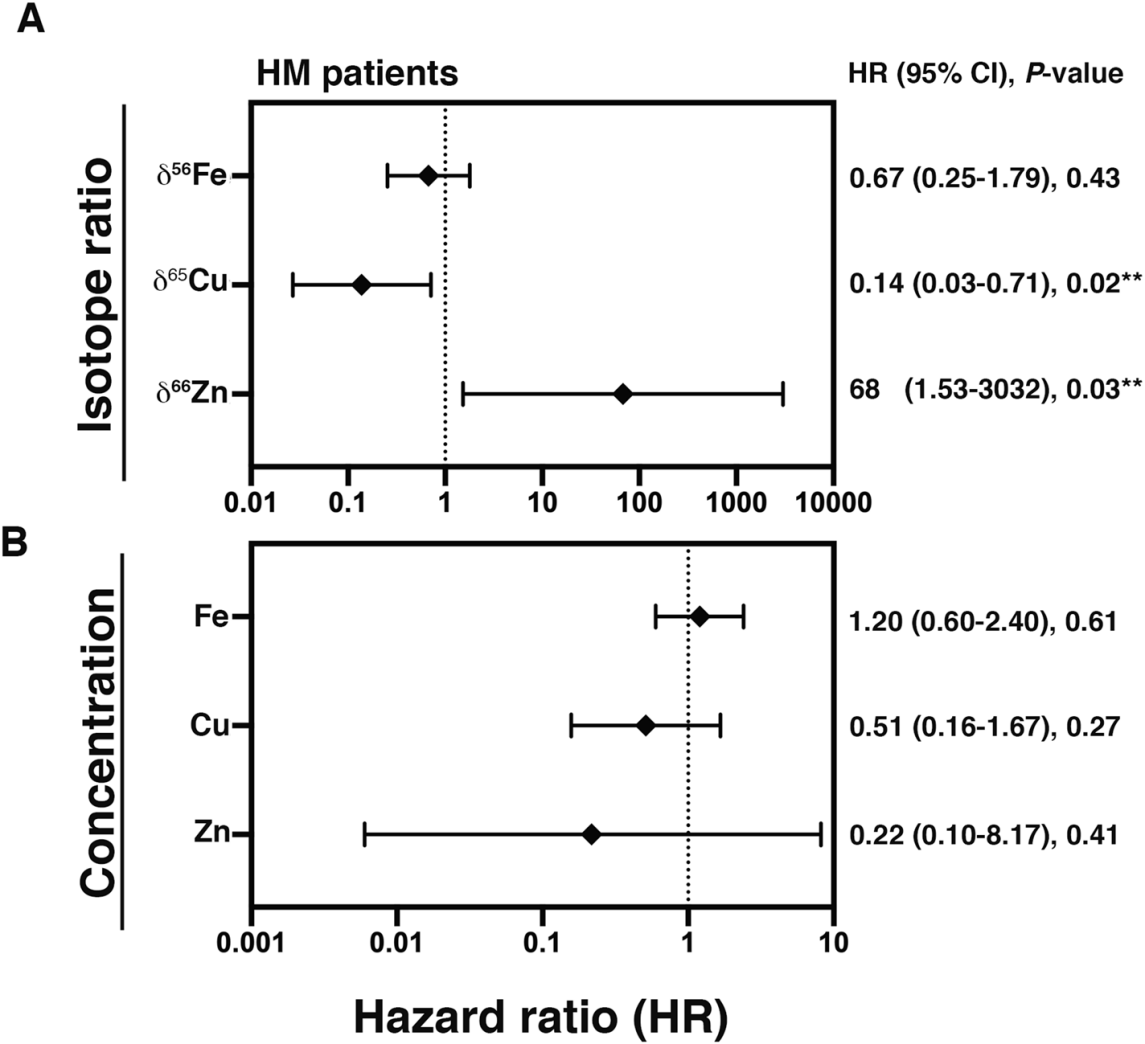
Mortality hazard ratios for a 5-years interval. Mortality hazard ratios (HR) were obtained by cox-regression analysis using the isotopic compositions (**A**) and the concentrations (**B**). **P < 0.05; 95% CI: 95% confidence interval.

Mortality hazard ratios (HRs) for 5-years survival were determined for Fe, Cu, and Zn isotope ratios and the corresponding concentrations (Fig. 3A,B, respectively). δ^66^Zn showed a HR higher than 1 (HR: 68, *P* = 0.03, Fig. 3A), suggesting a significantly higher risk of mortality in the case of increased δ^66^Zn values. δ^65^Cu showed a HR lower than 1 (HR: 0.14, *P* = 0.02, Fig. 3A), suggesting a significantly higher risk of mortality in the case of decreased δ^65^Cu values. On the other hand, δ^56^Fe (Fig. 3A), and Fe, Cu, Zn concentrations (Fig. 3B) did not show any significant influence on the HR.

**Figure 4:**
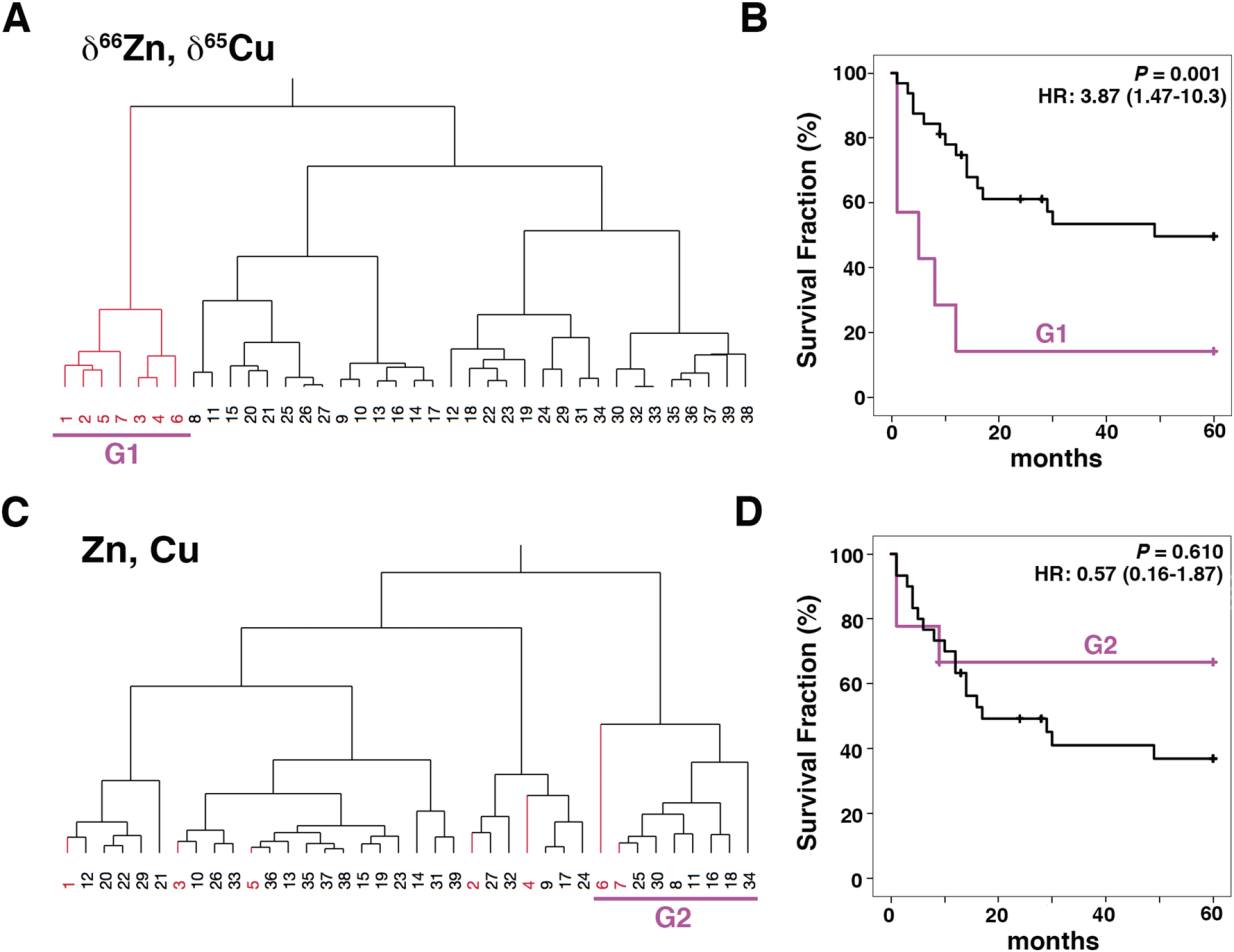
Survival analysis based on the combination of δ^65^Cu and δ^66^Zn values. (**A**) Hierarchical clustering analysis classified HM patients into two groups (G1 and the other group) using the δ^65^Cu and δ^66^Zn values. (**B**) Survival curves for G1 and the other group obtained by Kaplan-Meier analysis. G1 showed significantly poor survival from the early phase (*P* = 0.001). (**C**) Similar classification as in (A), but using the corresponding Cu and Zn concentrations. (**D**) HM patients were classified into two groups (G2 and the other group). Survival curves for G2 and the other group obtained by Kaplan–Meier analysis. The two groups did not show a significant difference. Patients are numbered according to the δ^66^Zn value (descending order); red coloured numbers correspond to patients in G1; HR, hazard ratio with 95% confidence interval.

We next examined survival curves using the combination of δ^65^Cu and δ^66^Zn data, both of which showed a significant influence on the HR. Hierarchical clustering analysis (HCA) was applied for grouping of the patients. HCA using the δ^65^Cu and δ^66^Zn values classified the HM patients into two groups at the highest hierarchy (G1 and the other group, see Fig. 4A). G1 was formed by patients showing high δ^66^Zn and low δ^65^Cu values compared to controls and it showed significantly poorer survival (HR at 60 months: 3.87, *P* = 0.001, Fig. 4B). Most patients in G1 died within 15 months (HR at 15 months: 5.12, 95% CI:1.81–14.47). These data suggest that G1 showed a high risk of mortality in an early phase. A similar approach was performed using the Cu and Zn concentrations (Fig. 4C,D); however, there was no significant difference between the G2 group and the other group found upon HCA (Fig. 4D).

The G1 group identified on the basis of isotope ratios was not significantly different from the other group in terms of disease type, age, and gender (Fig. S2). These data suggest that poor survival within G1 was not dependent on any difference related to disease type, age or gender and thus, they indicate the potential capability of the Cu and Zn isotopic compositions as prognostic factors in HM.

### Correlations of the metal isotope ratios with laboratory test values

**Figure 5:**
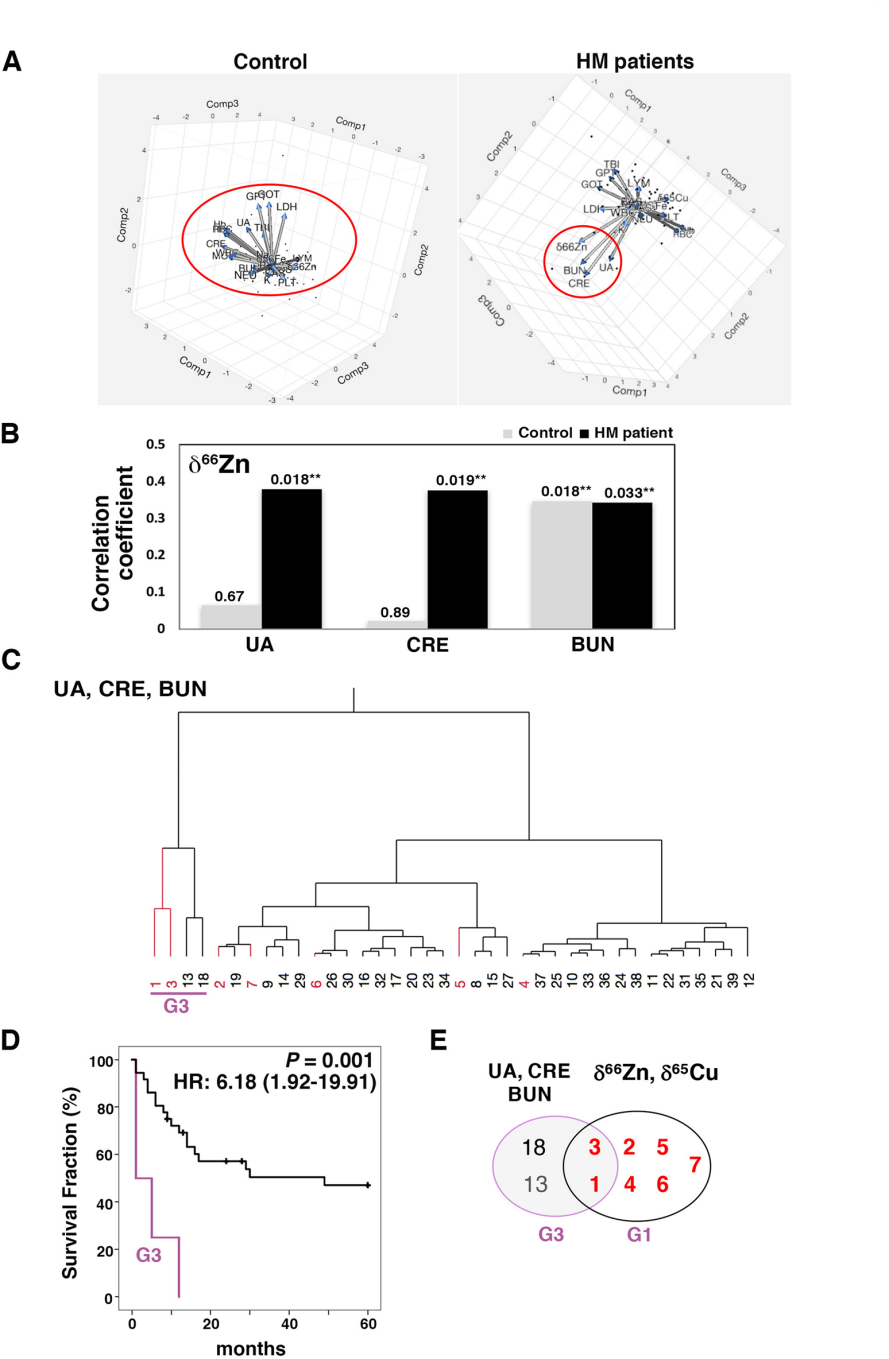
Correlations between the isotopic compositions and laboratory test values. (**A**) Principal component analysis (PCA) using the isotopic compositions and laboratory test values. PCA was performed using JMP (version 13.2.0, SAS Institute inc., Cary NC, USA). The red circle indicates a characteristic distribution of items in controls or HM patients (Adobe Photoshop CC, version 18.1.6, Adobe System Inc., CA, USA). (**B**) Correlations between the Zn isotopic composition and renal function parameters. The values above the bars correspond to the *P*-values for each correlation. Black bars: values for HM patients; grey bars: values for healthy controls, *P < 0.05. (**C**) Hierarchical clustering analysis using UA, CRE, BUN (renal function) classified HM patients into two groups (G3 and the other group). (**D**) Survival curves for G3 and the other group were obtained by Kaplan-Meier analysis. All patients in G3 died within 15 months, and G3 showed a significantly poor survival (*P* = 0.001). (**E**) Venn diagrams illustrating the partial overlap between G3 and G1. Two patients in G3 were also included in G1. Patients are numbered according to the δ^66^Zn value (descending order); red coloured numbers correspond to patients in G1. HR, hazard ratio with 95% confidence interval.

To understand the mechanisms affecting the isotope ratios, thus rendering them potentially useful for prognosis in HM, we next carried out Principal Component Analysis (PCA) using the isotope ratios and laboratory test values. The PCA biplot showed arrows of varied directions and lengths (Fig. 5A). Notably, δ^66^Zn formed a subgroup with three major laboratory test values related to renal function (uric acid concentration UA, creatinine concentration CRE and blood urea nitrogen concentration BUN) in HM patients, and the direction of the arrows was extended in the same direction (Fig. 5A, right). These data suggest a positive correlation between δ^66^Zn and renal parameters. On the other hand, the δ^66^Zn in controls did not lead to the formation of any obvious subgroup (Fig. 5A, left). Spearman’s rho test confirmed that δ^66^Zn correlated with UA, CRE and BUN (Fig. 5B, *P* < 0.05). These correlations became remarkably significant in the HM patients, whereas BUN was significantly correlated with δ^66^Zn in both HMs and controls. δ^66^Zn also correlated significantly with two laboratory test values related to anemia, *i.e.* the hematocrit level Ht and the hemoglobin level Hb, in HM patients (Spearman’s rho test, *P* = 0.007 and *P* = 0.006, respectively, Fig. S3.A). δ^56^Fe correlated with the platelets number PLT, which provides information on the bleeding tendency, and is often enhanced in leukemia (*P* = 0.03, Fig. S3A). In contrast, Fe, Cu and Zn concentrations did not show any significant correlation with renal function nor anemia in HM, while in the controls, these concentrations showed a correlation with the anemia-related values (RBC, Ht, Hb) and PLT (Fig. S3B).

We next examined to what extent those laboratory parameters that correlated with the isotope ratios could contribute to prognosis in HM and were related to G1. HCA using UA, CRE, BUN values classified HM patients into two groups (G3 and the other group, see Fig. 5C). All patients in G3 died within 15 months, and G3 showed a significantly poorer survival (HR 6.18, *P* = 0.001, Fig. 5D). In fact, G3 patients showed extremely poor results for CRE (Arrowheads, Fig. S4). These data were consistent with previous findings that identified CRE as a prognostic marker in HM patients^26–29^. Notably, only two patients in G3 also belonged to G1 (Fig. 5E). A similar approach was performed using Ht, Hb, PLT (Fig. S5A), and 6 or 20 of the laboratory test values (see Figs. S5B and S5C). There was no significant difference in survival between each of these clustering groups (G4, G5, G6) and the corresponding other group (Fig. S5A–C, right). These data suggest that only renal parameters including a well-known prognostic marker could reveal a risk group of early mortality (G3) that partially overlapped with G1.

### Time-dependent ROC curves for δ^65^Cu and δ^66^Zn

**Figure 6:**
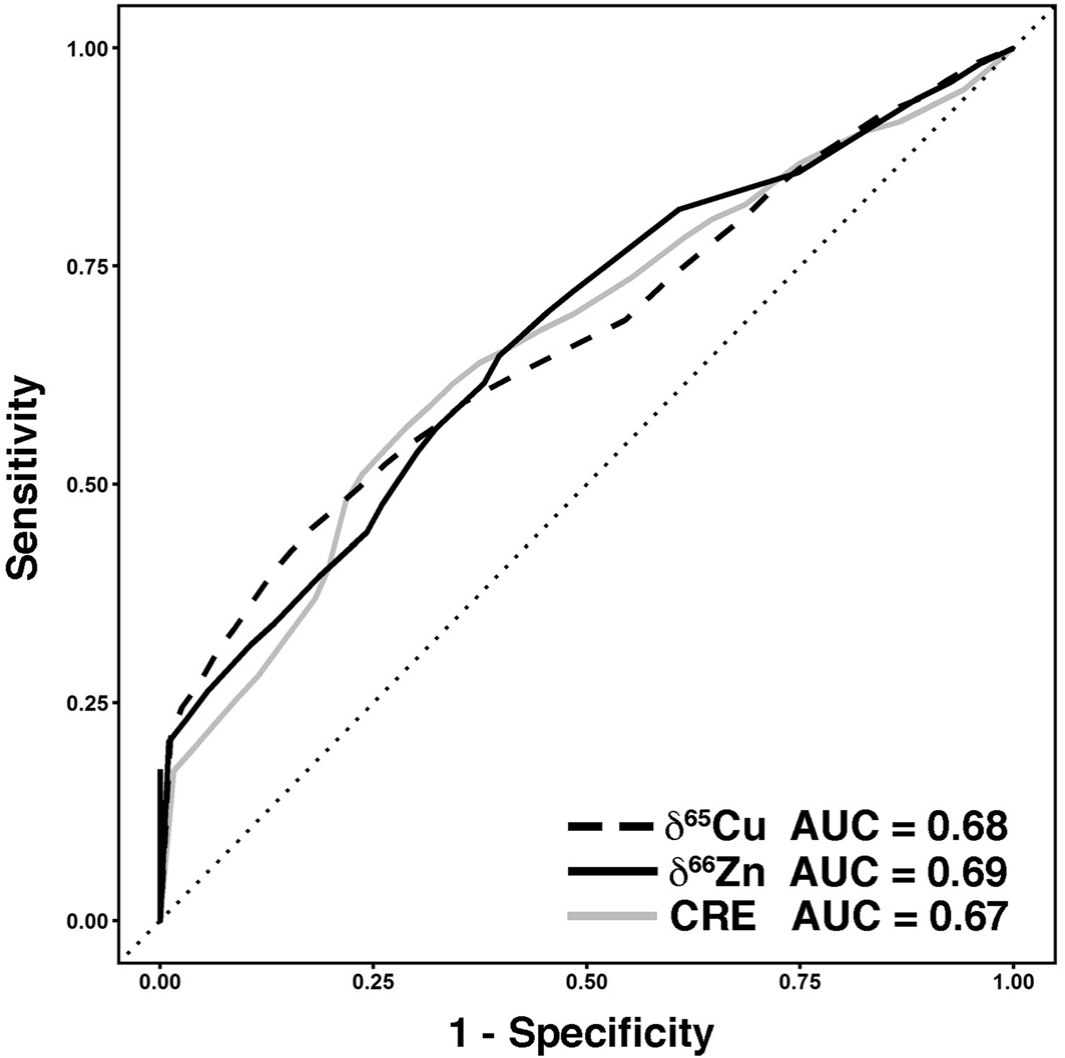
Time-dependent receiver operating characteristic (ROC) curve for δ^65^Cu, δ^66^Zn and CRE (creatinine), a well-known prognostic factor in HM; AUC, area under the curve.

To understand the capability of the δ^65^Cu or δ^66^Zn values as potential new prognostic factors of HMs, we next examined the corresponding time-dependent receiver operating characteristic (ROC) curves. The δ^65^Cu or δ^66^Zn value shows a similar area under the curve (AUC) as does CRE, a well-known prognostic factor in HM^26–29^ (Fig. 6).

## Discussion

Biomarkers allowing diagnosis in an early stage of a disease, and prognostic markers for predicting a patient’s survival are of outmost importance. To date, differences in the isotopic compositions of essential metals (Cu and Ca) in serum/red blood cells have been shown between cancer patients and controls^12, 13, 30^ and although the mechanisms or processes inducing these isotopic effects still have to be elucidated in detail, some interpretations have already been given. It has been suggested that the low serum δ^65^Cu values may be associated to changes in metalloproteins and/or reallocation of the Cu bound to these proteins^13^. Heavier isotopes are preferentially bound to amino acids with harder ligands, involving metal binding to nitrogen and oxygen (e.g., histidine and phosphate, respectively), while the lighter isotopes prefer binding to softer ligands, involving sulphur (such as cysteine and glutathione). Bonds involving the reduced metal form tend to be enriched in the light isotope^31^. These findings suggested that alterations in the natural isotopic compositions are associated with the development of diseases involved in the disruption of metal homeostasis. In this study, we have demonstrated the association between the isotopic compositions of Cu and Zn and prognosis for HM patients.

The plasma Zn isotopic composition of the HM patients was heavier than that of controls. In HM patients, δ^66^Zn values were correlated with renal dysfunction via three major test values, and with anemia and bleeding-related values, which are typically affected in HMs^32^. Notably, CRE is a well-known prognostic factor of HM^26–29^. In fact, CRE showed a significant mortality HR by COX regression analysis (HR 2.49: 95% CI, 1.30–4.76, *P* = 0.006). Additionally, anemia and the bleeding tendency are aggravating factors for HM prognosis. These correlations may at least partially explain why δ^66^Zn indicates the risk of mortality.

On the other hand, we could not find out any relation between δ^65^Cu values and laboratory test values. The Warburg effect has been proposed as an explanation for the light plasma/serum Cu isotopic composition in malignancy patients: a preferential chelation of the heavy ^65^Cu isotope in tumour cells, with the subsequent preferential release of the light ^63^Cu isotope into plasma or serum^12^, which was reported for various types of malignancies^12, 13^, and is consistent with our work on HM. Notably, although HM is a ‘non-solid tumour type’, it also causes the plasma Cu isotopic composition to be light, as was established for serum in solid tumours. As a result, the origin of this observation might be more complex than preferential ^65^Cu uptake in the solid tumour and preferential excretion of ^63^Cu into the blood stream. The enrichment in the light ^63^Cu isotope in plasma could be related to tumour cell proliferation. However, we could not see any correlation between the δ^65^Cu value and increased white cell numbers (WBC, Blasts, etc.) due to tumorigenesis in HM, nor between the δ^65^Cu value and UA or lactate dehydrogenase LDH levels, which are often influenced by tumour cell proliferation in HM. While leukemia results in abnormal cells in peripheral blood, lymphoma (NHL) and myeloma (MM) are mainly located at lymph nodes and in bone marrow, respectively. Therefore, next to specific cells in peripheral blood, also cells in bone marrow or lymph nodes that chelate the heavy ^65^Cu isotope, may play an important role. Further studies are necessary to reveal the mechanism of decreased δ^65^Cu values in these malignancies.

Notably, our study demonstrated that the combination of decreased δ^65^Cu and increased δ^66^Zn values, corresponding to the G1 group in this study, showed significantly poorer survival from the early phase. G1 partially overlapped with G3 with high risk of mortality due to deteriorated renal function. However, the rest of the HM patients in the G1 group formed a unique cohort revealed by Cu and Zn isotope ratios only. In fact, most of the patients in G1 had a similar range of renal function parameters as the rest of the HM patients, except for the two cases in G3 (Figs. 5C,E and S4). On the other hand, G1 showed remarkably worse laboratory test values related to anemia and bleeding tendency compared to the rest of the HM patients (Fig. S4). However, critical survival of G1 could not be explained by anemia, increased bleeding tendency and a minor impact on renal dysfunction only. There have to be further so far unrevealed mechanisms explaining the decreased δ^65^Cu and increased δ^66^Zn values. Further clinical studies are required to understand the mechanisms for decreased δ^65^Cu and increased δ^66^Zn values that predict poor survival in HM. It was noticeable that the δ^65^Cu or δ^66^Zn values showed a similar time-dependent ROC curve to CRE. Overall, our data suggest that δ^65^Cu or δ^66^Zn contained equally significant, but different prognostic information, which matched that offered by the well-known prognostic factor CRE. The δ^65^Cu and δ^66^Zn values could be interesting new candidates as prognostic factors in HM patients.

## Conclusions

High-precision isotopic analysis using multi-collector ICP-mass spectrometry revealed significant changes in the isotopic compositions of Cu (isotopically lighter) and Zn (isotopically heavier) in the plasma of HM patients. Patients with decreased δ^65^Cu and increased δ^66^Zn showed significantly poorer survival from the early phase, forming a unique cohort not revealed based on laboratory test values. The assessment of the HRs and the time-dependent ROC curves suggest that the δ^65^Cu and δ^66^Zn values are useful for the prediction of survival in HM patients. This study was exploratory in nature and future studies with a larger number of patients in a multi-center study are recommended.

## Materials and methods

### Patients

Patients suffering from HMs, defined according to the 2008 WHO classification^33^, were recruited on their first visit to the hospital of the National Center for Global Health and Medicine (NCGM) in Tokyo, Japan, from April 2008 to July 2009. Patients with chemotherapy at sampling were excluded. In total, 47 patients suffering from HMs were selected (Table 1). Patients were followed up from the first visit to April 2014 or to death for survival analysis. The median follow-up time was 33.3 months. A set of 22 basic laboratory tests, including complete blood counts (CBC), were carried out at the NCGM hospital. As controls, gender- and age-matched healthy cases, visiting for a regular medical check-up (as required by the national health insurance in Japan) between May 2009 and June 2009, were recruited. All samples were obtained following fasting.

### Laboratory tests

A total of 22 basic and CBC laboratory tests were completed in the routine medicine division of the NCGM hospital, including: counting the number of white blood cells (WBC), blast cells (BLAS), neutrophils (NEU), monocytes (MON), eosinophils (EOS), basophils (BAS), lymphocytes (LYM) and red blood cells (RBC) and the determination of the levels of hematocrit (Ht), hemoglobin (Hb), platelets (PLT), albumin (ALB), total bilirubin (TBI), aspartate aminotransferase (GOT), alanine aminotransferase (GPT), lactate dehydrogenase (LDH), urea nitrogen (BUN), creatinine (CRE), uric acid (UA), sodium (Na), potassium (K), and chloride (Cl).

### Sample preparation

Winged needle (MN-SVS23BS, Terumo, Tokyo, Japan) vacuum blood collection tubes (Venoject II VP-CW052K, Terumo, Tokyo, Japan) were used to collect 4 mL of peripheral blood. Laboratory tests followed the sampling immediately. The collection tubes were centrifuged at 250 × *g* for 20 min at 4 °C, after which the supernatant plasma fraction was collected in a centrifuge tube (15 mL, #2327-015, Iwaki, Tokyo, Japan). An aliquot of plasma was used for elemental analysis (at NCGM) and another aliquot was used for isotopic analysis (at Ghent University). Samples for isotopic analysis were stored in pre-cleaned Eppendorf tubes at − 20 °C and transported on dry ice.

Sample preparation for isotopic analysis was performed in a class-10 clean room (PicoTrace, Göttingen, Germany). Ultra-pure water acquired from a Milli-Q water purification system (Merck Millipore, Molsheim, France) was used throughout. *Pro-analysis* grade HNO_3_ (Chem-Lab, Zedelgem, Belgium) and HCl (Fisher Chemicals, Loughborough, UK) were further purified by subboiling distillation in a Savillex DST-4000 acid purification system (Savillex Corporation, Eden Prairie, MN, USA) prior to usage. The serum samples were digested with a mixture (4:1 v/v) of 14 M HNO_3_ and 9.8 M H_2_O_2_ (Sigma Aldrich, Belgium) kept at 110 °C for 18 h. The digests were evaporated to dryness and re-dissolved in 5 mL of a solution containing 8 M HCl and a small amount of H_2_O_2_ (~ 0.001%) for the sequential isolation of Cu, Fe and Zn via anion exchange chromatography using 1 mL of AG-MP1 resin. The Cu fraction was eluted using 9 mL of 5 M HCl +  ~ 0.001% H_2_O_2_, the Fe fraction using 7 mL of 0.6 M HCl and the Zn fraction using 7 mL of 0.7 M HNO_3_. The Cu fractions were subjected to a second column pass to ensure a Na/Cu ratio < 2 in all solutions^17, 22^. The pure fractions thus obtained were subjected to two steps of drying and re-dissolution in 14 M HNO_3_ to remove residual chlorides and, were finally re-dissolved in 0.5 mL of 0.42 M HNO_3_ for high-precision isotope ratio measurements using MC-ICP-MS. For elemental analysis, plasma samples were digested with 0.5 mL of 14 M HNO_3_ (Tamapure-AA-100, Tama Chemical Co. Ltd., Kanagawa, Japan) at 180 °C for 20 min using an ETHOS 1 microwave dissolution unit (Milestone, Shelton, CT, USA) and then diluted with Milli-Q water (Nihon Millipore, Tokyo, Japan) to a final volume of 5 mL.

### Determination of metal isotope ratios and concentrations

e, Cu and Zn isotope ratios were measured using a Neptune multi-collector inductively coupled plasma-mass spectrometry (MC-ICP-MS) instrument (ThermoScientific, Bremen, Germany). The bias caused by instrumental discrimination was corrected for by means of a combination of internal correction (internal standard) and external correction (external standard). Ni, Ga and Cu (Inorganic Ventures, VA, USA) were used as internal standard for Fe, Cu and Zn isotope ratio measurements, respectively. Internal correction was done according to the revised Russell’s law^34^. For external correction, the samples and the external standard were measured in a sample-standard bracketing approach (SSB). The concentrations of standards and samples were always matched within ± 5%. The reference materials Cu NIST SRM-976 (NIST, Gaithersburg, MD, USA), Fe IRMM-014 and Zn IRMM-3702 (IRMM, Geel, Belgium) were used as external isotopic standards. The isotope ratios were expressed in δ-values (in ‰), calculated as indicated in Eq. (1).

1 $$\delta^{a/b} X_{sample} = \left( {\frac{{^{a/b} R_{sample} }}{{^{a/b} R_{standard} }} - 1} \right) \times 1000$$

where a and b correspond to the mass numbers of the isotopes of interest, X is the target element and R an isotope ratio of the target element.

Fe, Cu and Zn standard solutions (Inorganic Ventures) that were previously characterized isotopically were measured every five samples for quality assurance/quality control (QA/QC) of the measurements. The δ-values (mean ± 2SE) obtained along this work were 0.23 ± 0.01‰ for δ^65^Cu (N = 53), 0.46 ± 0.02‰ for δ^56^Fe (N = 53), and − 7.04 ± 0.01‰ for δ^66^Zn (N = 40), which all agreed well with the data from previous studies^17, 21, 22^.

The expanded uncertainty, which characterizes the dispersion of the δ-values in the plasma samples, was 0.05‰ for the δ^65^Cu, 0.18‰ for the δ^56^Fe, and 0.06‰ for the δ^66^Zn.

Total concentrations of Fe, Cu and Zn were determined using ICP-AES (Optima 4300DV, PerkinElmer, Waltham, MA, USA). The signal at the optimal wavelength for each element (Fe: 259.939 nm; Cu: 327.393 nm; Zn: 213.857 nm) was used for quantification. Data were validated using a human serum reference material (Seronorm, Sero, Billingstad, Norway, Table S2).

### Statistical analysis

Data standardization, correlation analysis, cox regression and survival analysis were performed with SPSS (version 26; IBM, Armonk, NY, USA). The limit of significance for all analyses was set at *P* = 0.05. Group comparisons were carried out using the Kolmogorov–Smirnov test for continuous variables and the chi-square test for categorical variables using GraphPad Prism software (version 8; GraphPad Software Inc., San Diego, CA, USA). Principal component analysis (PCA) was performed using JMP (version 13.2.0; SAS Institute inc., Cary NC, USA). A score scatter plot was generated to obtain an overview of sample clustering and to detect potential outliers. Varimax rotation was applied for data interpretation. Correlation coefficients between parameters were assessed using the Spearman rank test. To examine correlation between survival and the elemental concentrations/isotope ratios, the hazard ratios (HR) with 95% confidence intervals (CI) were calculated by univariate and multivariate Cox regression analyses. The element concentration/isotope ratio was considered significant when the log-rank test *P*-value was < 0.05 in the univariate Cox regression analysis and was then selected for multivariate analysis. Hierarchical clustering analysis (HCA) was performed using Ward’s methods by JMP (version 13.2.0; SAS Institute inc. Cary, NC, USA), and validated using the R *hclust* function (version 3.6.3). The overall survival of each group was calculated using the Kaplan–Meier method. Two survival curves were compared by the Gehan–Breslow–Wilcoxon test. The Cox proportional hazard models were used to estimate HRs and 95% CIs for comparison of death event rates between G1 and the other groups. Multivariate Cox regression analysis was performed using the survival package (version 3.1.8) in the R language (version 3.5.0)^35^. Subsequently, the time-dependent receiver operating characteristic (ROC) curve analysis was conducted using R with the survival ROC package (version 1.0.3)^36^. The results were expressed as indicated in the STROBE guidelines.

### Ethical approval

This study was performed following the national regulations and institutional policies and was approved by the committees of the National Center for Global Health and Medicine (#NCGM-G-003014–00) in accordance with the Helsinki Declaration of the World Medical Association. All subjects provided informed consent.

## Supplementary information

Supplementary file1

## Data Availability

The authors state that there is not any restriction on the availability of materials or information.
