# Supplementary material for: Cu and Zn isotope ratio variations in plasma for survival prediction in hematological malignancy cases
Source: Sci Rep. 2020 Oct 2;10:16389. doi: 10.1038/s41598-020-71764-7 (PMC7532200; doi:10.1038/s41598-020-71764-7)
Supplement: Supplementary file 1 — Supplementary file1 [file 41598_2020_71764_MOESM1_ESM.docx]

# Cu and Zn isotope ratio variations in plasma for survival prediction in haematological malignancy cases

**Agustina A. M. B. Hastuti,*a Marta Costas-Rodríguez,*a Akihiro Matsunaga,*b Takayuki Ichinose, c Shotaro Hagiwara, d Mari Shimura,†b Frank Vanhaecke†a**

a Ghent University, Department of Chemistry, Atomic & Mass Spectrometry – A&MS research unit, Campus Sterre, Krijgslaan 281 – S12, 9000 Ghent, Belgium

b Department of Intractable Diseases, Research Institute, National Center for Global Health and Medicine, 1-21-1 Toyama, Shinjuku, Tokyo 162-8655, Japan

c Inorganic Analysis Laboratories, Toray Research Center Inc., Otsu, Shiga 520-8567, Japan d Division of Hematology, Internal Medicine, Hospital, National Center for Global Health and Medicine, Shinjuku, Tokyo, Japan/ Current address: Division of Hematology, School of Medicine, Tokyo Women’s Medical University, Shinjuku, Tokyo 162-8666, Japan

* Authors contributed equally to this work.

† Corresponding authors’ e-mails: mshimura@ri.ncgm.go.jp; [frank.vanhaecke@ugent.be](mailto:frank.vanhaecke@ugent.be)

# Electronic supplementary information

Tables S1 and S2

Figures S1-S5

**Table S1.** Plasma Cu, Fe and Zn isotopic compositions of Japanese healthy controls and HM patients. The expanded uncertainties for the δ^56^Fe, δ^65^Cu and δ^66^Zn values are 0.05, 0.18 and 0.06 ‰, respectively.

| **ID** | **Healthy controls** | | | | | **ID** | **HM patients** | | | | |
| --- | --- | --- | --- | --- | --- | --- | --- | --- | --- | --- | --- |
|  | **Gender** | **Age** | **δ^56^Fe** | **δ^65^Cu** | **δ^66^Zn** |  | **Gender** | **Age** | **δ^56^Fe** | **δ^65^Cu** | **δ^66^Zn** |
| *C01#* | F | 52 | -2.83 | -0.28 | 0.25 | *HM01#* | F | 40 | -2.73 | -0.22 | 0.32 |
| *C02#* | F | 50 | -2.51 | -0.46 | 0.32 | *HM02#* | M | 59 | -2.24 | -0.66 | 0.22 |
| *C03#* | F | 63 | -1.49 | -0.44 | 0.28 | *HM03#* | F | 54 |  | -0.01 | 0.26 |
| *C04#* | F | 66 | -2.88 | -0.26 | 0.21 | *HM04#* | F | 57 | -3.27 | -0.69 | 0.41 |
| *C05#* | F | 45 | -2.26 | -0.37 | 0.19 | *HM05#* | F | 63 | -3.00 | -0.98 | 0.25 |
| *C06#* | F | 62 | -2.40 | -0.39 | 0.38 | *HM06#* | M | 65 | -2.52 | -0.73 | 0.57 |
| *C07#* | M | 66 | -2.79 | 0.11 | 0.26 | *HM07#* | F | 65 | -3.85 | -0.10 | 0.33 |
| *C08#* | F | 39 | -3.34 | -0.08 | 0.19 | *HM08#* | F | 40 | -1.80 | -0.65 | 0.25 |
| *C09#* | F | 32 | -3.14 | -0.21 | 0.24 | *HM09#* | F | 83 | -2.53 | -0.61 | 0.30 |
| *C10#* | F | 78 | -2.70 | -0.15 | 0.37 | *HM10#* | F | 56 | -3.66 | 0.20 | 0.41 |
| *C11#* | M | 45 | -3.35 | -0.11 | 0.13 | *HM11#* | M | 18 | -3.19 | 0.10 | 0.44 |
| *C12#* | M | 62 | -2.89 | -0.26 | 0.25 | *HM12#* | F | 63 | -2.57 | -0.53 | 0.12 |
| *C13#* | F | 66 | -2.54 | -0.31 | 0.33 | *HM13#* | M | 80 | -3.39 | -0.67 | 0.35 |
| *C14#* | F | 65 | -2.95 | -0.38 | 0.25 | *HM14#* | M | 71 | -2.79 | -0.62 | 0.55 |
| *C15#* | F | 63 | -2.81 | -0.26 | 0.20 | *HM15#* | F | 79 | -2.78 | -0.30 | 0.22 |
| *C16#* | F | 43 | -2.51 | -0.26 | 0.24 | *HM16#* | F | 61 | -2.68 | -0.42 | 0.42 |
| *C17#* | F | 58 | -2.62 | -0.09 | 0.27 | *HM17#* | M | 80 | -3.03 | -0.35 | 0.39 |
| *C18#* | F | 55 | -3.21 | -0.30 | 0.28 | *HM18#* | M | 48 | -3.52 | -0.20 | 0.25 |
| *C19#* | M | 54 | -3.18 | 0.09 | 0.22 | *HM19#* | M | 70 | -3.19 | -0.97 | 0.48 |
| *C20#* | M | 60 | -2.96 | -0.62 | 0.42 | *HM20#* | F | 39 | -2.59 | -0.44 | 0.37 |
| *C21#* | M | 84 | -2.67 | -0.06 | 0.37 | *HM21#* | F | 54 | -2.85 | -0.09 | 0.26 |
| *C22#* | M | 66 | -2.89 | -0.46 | 0.28 | *HM22#* | M | 73 | -3.42 | -0.02 | 0.27 |
| *C23#* | F | 53 | -2.46 | -0.01 | 0.22 | *HM23#* | M | 68 | -2.70 | -0.46 | 0.22 |
| *C24#* | F | 62 |  | -0.07 | 0.22 | *HM24#* | F | 54 | -2.30 | -0.47 | 0.33 |
| *C25#* | F | 70 |  | -0.23 | 0.20 | *HM25#* | M | 61 | -3.18 | -0.42 | 0.17 |
| *C26#* | F | 73 | -2.53 | -0.16 | 0.14 | *HM26#* | M | 37 | -3.08 | -0.12 | 0.38 |
| *C27#* | F | 56 | -2.97 | -0.18 | 0.22 | *HM27#* | F | 73 | -3.31 | -0.28 | 0.14 |
| *C28#* | M | 58 | -2.96 | -0.14 | 0.19 | *HM28#* | F | 73 | -3.09 | -0.41 | 0.20 |
| *C29#* | F | 29 | -2.43 | -0.21 | 0.19 | *HM29#* | M | 51 | -3.77 | -0.81 | 0.51 |
| *C30#* | M | 78 | -2.26 | -0.31 | 0.29 | *HM30#* | M | 56 | -2.88 | -0.45 | 0.39 |
| *C31#* | M | 62 | -2.99 | -0.26 | 0.45 | *HM31#* | M | 46 | -3.06 | -0.65 | 0.29 |
| *C32#* | M | 41 | -3.16 | -0.15 | 0.19 | *HM32#* | M | 73 | -2.61 | -0.86 | 0.28 |
| *C33#* | M | 75 | -2.91 | -0.70 | 0.29 | *HM33#* | M | 41 | -3.05 | -0.08 | 0.26 |
| *C34#* | M | 55 | -2.88 | -0.05 | 0.28 | *HM34#* | F | 62 | -2.56 | -0.33 | 0.37 |
| *C35#* | M | 64 | -3.83 | -0.31 | 0.17 | *HM35#* | F | 23 | -3.34 | -0.41 | 0.50 |
| *C36#* | F | 48 | -2.96 | 0.05 | 0.18 | *HM36#* | F | 45 | -3.08 | -0.39 | 0.44 |
| *C37#* | M | 63 | -2.65 | -0.29 | 0.23 | *HM37#* | M | 66 | -2.07 | -0.89 | 0.56 |
| *C38#* | M | 41 | -2.87 | 0.03 | 0.25 | *HM38#* | M | 55 | -2.95 | -0.55 | 0.55 |
| *C39#* | M | 74 | -2.05 | -0.26 | 0.32 | *HM39#* | M | 41 | -2.58 | -0.29 | 0.22 |
| *C40#* | F | 75 | -2.45 | -0.10 | 0.26 |  |  |  |  |  |  |
| *C41#* | M | 74 | -2.85 | -0.31 | 0.11 |  |  |  |  |  |  |
| *C42#* | M | 62 | -2.54 | -0.02 | 0.06 |  |  |  |  |  |  |
| *C43#* | M | 41 | -3.11 | -0.14 | 0.06 |  |  |  |  |  |  |
| *C44#* | F | 55 | -2.52 | -0.40 | 0.35 |  |  |  |  |  |  |
| *C45#* | M | 64 | -2.47 | -0.15 | 0.31 |  |  |  |  |  |  |
| *C46#* | M | 57 | -3.28 | -0.06 | 0.15 |  |  |  |  |  |  |
| *C47#* | M | 66 |  | -0.07 | 0.25 |  |  |  |  |  |  |


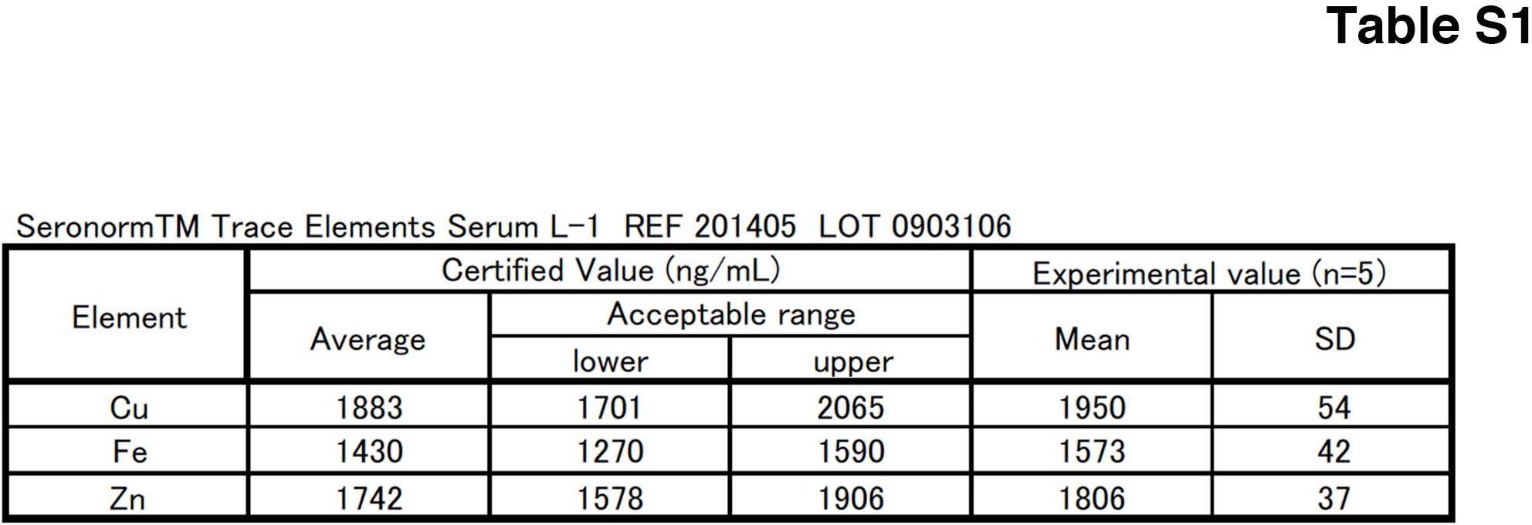
**Table S2.** Metal concentrations in Seronorm (Sero, Norway) human serum reference material obtained *via* ICP-AES. Data were within certified ranges.

**Fig. S1**

**Figure S1.** Comparison of the Fe, Cu and Zn isotopic compositions (isotope ratios expressed as delta values, ‰) for Japanese healthy controls (this study) and reference populations from other geographical origins. The box-plots were created using SigmaPlot (version 13, Systat Software, Inc, San Jose, CA, USA) and compile the median, quartiles and extreme values; individual dots are outliers. δ^56^Fe values in females (A), δ^56^Fe values in males (B), δ^65^Cu values (C) and δ^66^Zn values (D) in both males (M) and females (F). Filled (red) box plots correspond to plasma and empty (blue) box plots to serum. Reported data were compiled from references: von Blanckenburg, F., Oelze, M., Schmid, D.G., van Zuilen, K., Gschwind, H.P., Slade, A.J. *et al.* *Metallomics* **6**, 2052-2061 (2014); Hotz, K. & Walczyk, T*. J. Biol. Inorg. Chem.* **18**, 1-7 (2013); Hastuti A.A.M.B., Costas-Rodríguez, M., Anoshkina, Y., Parnall, T., Madura II, J.A. & Vanhaecke, F*.* *Anal. Bioanal. Chem.* **412**, 727-738 (2020); Albarède, F., Telouk, P., Lamboux, A., Jaouen, K. & Balter, V. *Metallomics* **3**, 926-933 (2011) and Lauwens, S., Costas-Rodríguez, M., Delanghe J., Van Vlierberghe, H. & Vanhaecke, F. *Talanta* **189**, 332-338 (2019). The δ^66^Zn data from Albarede *et al.* were recalculated relative to the IRMM-3702 isotopic reference material.

**Fig. S2**


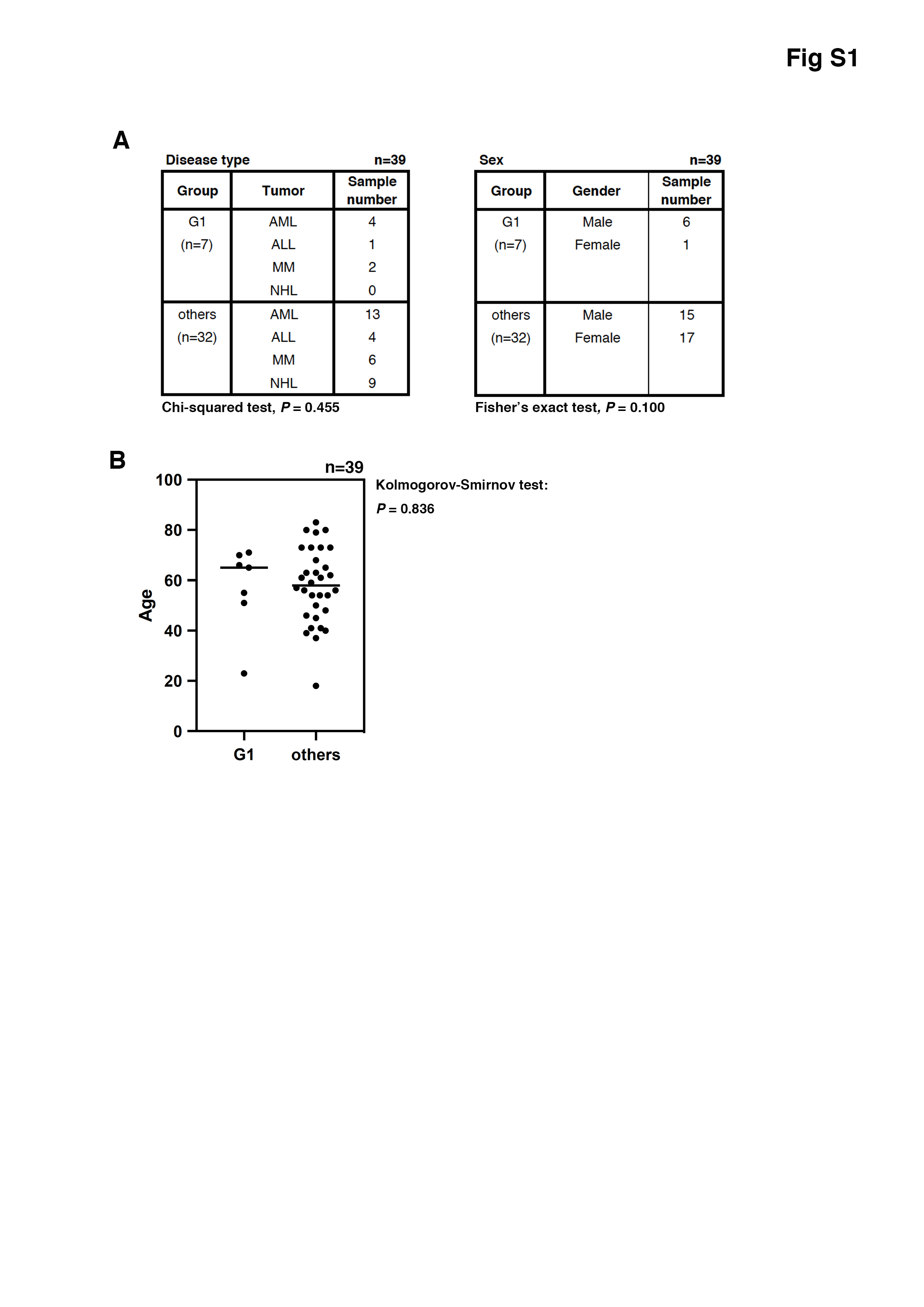


**Figure S2.** Classification of G1 and other patients by disease type, age and gender. Patient classification was performed by hierarchical clustering analysis. (A and B). G1 group was not significantly different in terms of disease type, gender, and age.

**Fig. S3**


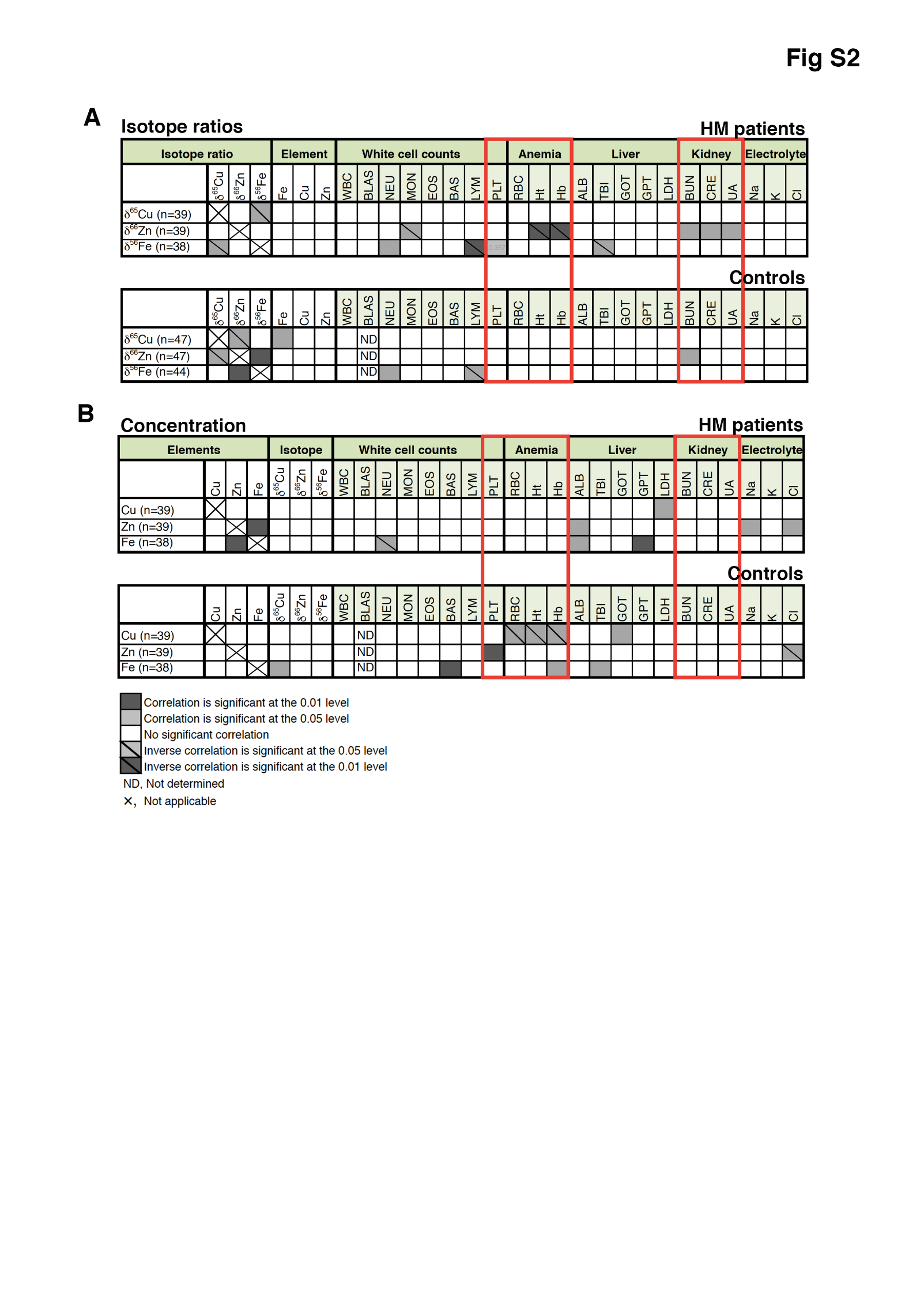


**Figure S3.** Correlation between the isotopic compositions and the concentrations of Fe, Cu, Zn with laboratory test vales. Relevant values are shown in Fig. 5 **A:** Correlations between Fe, Cu and Zn isotopic compositions and laboratory test values. **B**: Correlations between Fe, Cu and Zn concentrations and laboratory test values. Red rectangles indicate notable changes in the correlations between HM patients and controls. The Spearman rank test was used to examine the correlations.

**Fig. S4**


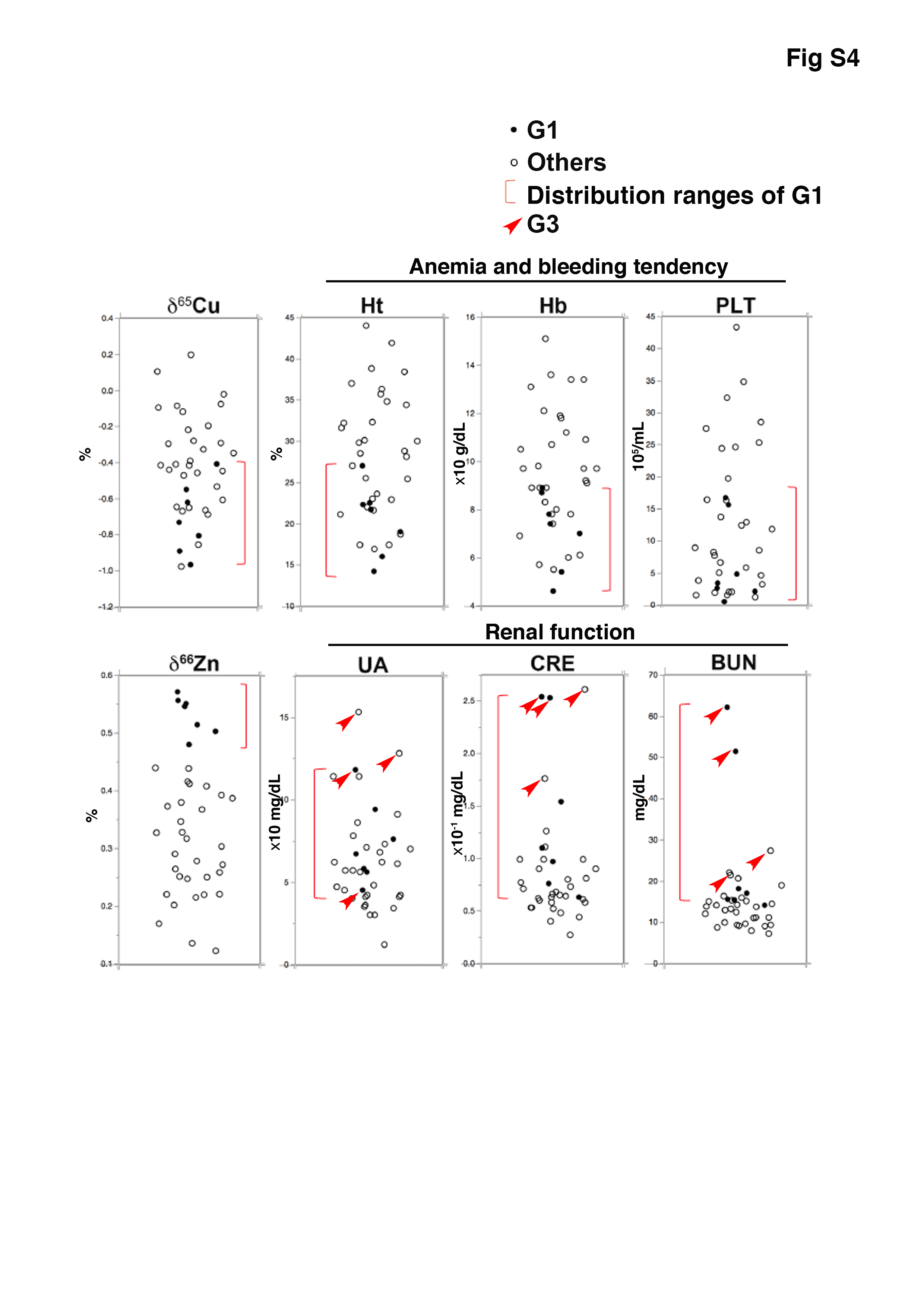


**Figure S4.** Distribution of laboratory test values for G1 (black solid circles) and the other group of HM patients (open circles). HM patients in G1 tend to show relatively lower values of Ht, Hb, and PLT (anemia and bleeding tendency), and similar ranges for BUN, CRE, and UA (renal function) to the other group, except for two cases in G3 (Fig. 5C and 5E). The red bracket indicates the range of the G1 values. Arrowheads indicate HM patients in G3.

**Fig. S5**

**
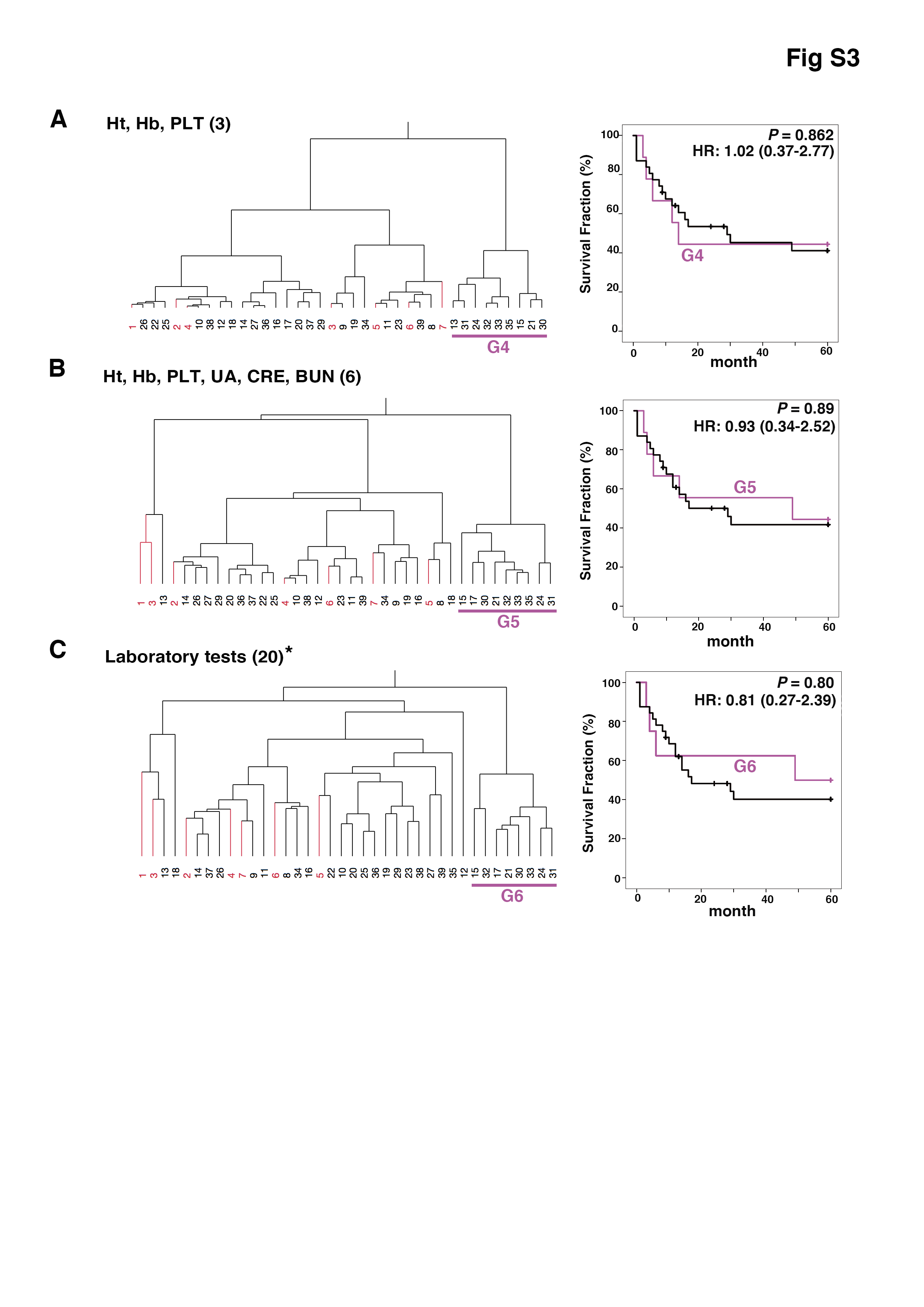
**

**Figure S5.** Classification of HM patients using laboratory test values and corresponding survivals. **A**: Left, hierarchical clustering analysis using Ht, Hb, and PLT (anemia and bleeding tendency) classified HM patients into two groups (G4 and the other group). Right, survival curves for G4 and the other group were obtained by Kaplan-Meir analysis. G4 did not show a significant difference. **B**: Similar analysis as in A using 6 laboratory test values (Ht, Hb, PLT, UA, CRE, BUN) and **C**: using 20 laboratory test values. HM patients were classified into two groups (G5 or G6 and the other group). G5 and G6 did not show a significant difference. *, ALB and TBI were excluded due to missing values. Patients numbering according to δ^66^Zn value – descending order; red colour numbers correspond to patients in G1 group. HR, hazard ratio with 95% confidence interval.
